# Prediction of Mechanical Twinning in Magnesium Silicate Post-Perovskite

**DOI:** 10.1038/s41598-017-18018-1

**Published:** 2017-12-15

**Authors:** Philippe Carrez, Alexandra M. Goryaeva, Patrick Cordier

**Affiliations:** Univ. Lille, CNRS, INRA, ENSCL, UMR 8207 UMET - Unité Matériaux et Transformations, F-59000 Lille, France

## Abstract

The plastic properties of MgSiO_3_ post-perovskite are considered to be one of the key issues necessary for understanding the seismic anisotropy at the bottom of the mantle in the so-called D” layer. Although plastic slip in MgSiO_3_ post-perovskite has attracted considerable attention, the twinning mechanism has not been addressed, despite some experimental evidence from low-pressure analogues. On the basis of a numerical mechanical model, we present a twin nucleation model for post-perovskite involving the emission of 1/6 <110> partial dislocations. Relying on first-principles calculations with no adjustable parameters, we show that {110} twin wall formation resulting from the interaction of multiple twin dislocations occurs at a twinning stress comparable in magnitude to the most readily occurring slip system in post-perovskite. Because dislocation activities and twinning are competitive strain-producing mechanisms, twinning should be considered in future models of crystallographic preferred orientations in post-perovskite to better interpret seismic anisotropy in the lowermost lower mantle.

## Introduction

Seismic anisotropy is one of our major sources of information about the dynamic processes and flow in the Earth’s mantle. In contrast to the bulk of the lower mantle, which appears to be mostly isotropic, the lowermost lower mantle exhibits strong seismic anisotropy and major heterogeneities. In particular, distinct anisotropy signatures are found in regions thought to be associated with cold downwelling^1,2^. The discovery in 2004 that bridgmanite, the magnesium silicate with perovskite structure which is the main constituent of the lower mantle, is not stable at pressures comparable to those of the D” layer and transforms at *c.a*. 120 GPa into a distinct structurally anisotropic phase, named post-perovskite^3^, has attracted considerable attention. The potential link between this new phase and the seismic anisotropy in the lowermost lower mantle received more support when it was established that the post-perovskite phase can form only in a relatively cold mantle^4^. The high-pressure magnesium silicate post-perovskite phase exhibits an orthorhombic layered crystal structure of space group *Cmcm* with strongly different lattice parameters^3^. Exhibiting layers of SiO_6_ octahedrons parallel to {010}, the structure is thus highly anisotropic with such a structural characteristic being potentially related to the strong seismic anisotropy of D”. To further establish the role of post-perovskite and to ultimately decipher the flow patterns at the base of the mantle, it is necessary to understand how crystal preferred orientations (CPOs) develop in this phase during plastic flow^5^. Given the very high-pressure required to stabilize the magnesium silicate post-perovskite, only a few set of experiments have been conducted on this phase^6–8^ and most experiments have been performed on analogue materials with the same crystal structure^9–11^, but stable at lower pressures. This includes calcium iridate (CaIrO_3_), which is stable at ambient pressure^12–15^. Unfortunately, all these experiments have led to conflicting results, possibly because of textures inherited from phase transformations^11,12,16^ and differences in the crystal chemistry of the analogue materials^8,17,18^. Given the formidable difficulties of deformation experiments under very high pressures, numerical modelling currently represents a very attractive alternative. Using atomic-scale modelling of dislocations^19–21^, we have shown that shearing the post-perovskite structure occurs easily along the shortest [100] lattice repeat in the Mg-O layer (010) plane, with a lattice friction of 2 GPa^20^. The other dense direction in this plane, [001], is the second easiest^21^ (ca. 3 GPa). However, shearing the Si-bearing layers appears to be much more difficult, because of the breaking of the strong Si-O bonds. Indeed, the lattice friction opposed to [100](001) is on the order of 17 GPa^19^. On the basis of these results, strong CPO along (010) is thus expected in post-perovskite. However, this cannot be the end of the story because a crystalline aggregate must sustain some strain components along the three directions of space to satisfy strain compatibility. Therefore, to provide reliable models of crystal preferred orientations and hence of seismic properties, it is necessary to understand which deformation mechanisms are active in this structure.

Mechanical twinning is a deformation mechanism that has received little attention despite microscopic observations of its occurrence in deformed CaIrO_3_ post-perovskite^13,14^. In this paper, we show that [010] dislocations are not stable in MgSiO_3_ post-perovskite, leading to partial dislocations that may be linked to mechanical twinning. Hence we present a hierarchical numerical model of the mechanical twinning in MgSiO_3_ post-perovskite at 120 GPa^3^, which is compared with the dislocation activity to assess its possible relevance in plastic flow and CPO development in post-perovskite in the lowermost lower mantle.

## Results

MgSiO_3_ post-perovskite phase exhibits an orthorhombic structure. The computed lattice parameters at a pressure of 120 GPa are *a* = 2.47 Å, *b* = 8.11 Å and *c* = 6.14 Å. The very strong anisotropy of the unit cell and the characteristic layering of the structure have led to a focus on plastic shear along [100]. Not surprisingly, this shear is very easy to produce along the magnesium layer in the (010) plane^19,20^. The second easiest slip system also corresponds to shear in (010) but along the [001] direction^21^. Shear along [010] raises some questions, because it would require activating dislocations with a very large Burgers vector. This is usually unfavourable since the elastic energy of a dislocation scales to the square of the modulus of the Burgers vector. The classical Frank criteria show that [010] dislocations are not stable and decompose into two ½<110> dislocations because *b* ^2^_[010]_ > 2x*b* ^2^_½<110>_. Indeed, in the orthorhombic C-lattice, ½<110>, with a length 4.2 Å, is one of the shortest lattice repeats of the structure and consequently represents another potential Burger vectors. Therefore, in this work, we start by addressing the properties of ½<110> dislocation cores.

### ½<110> dislocation cores in MgSiO_3_ post-perovskite

**Figure 1:**
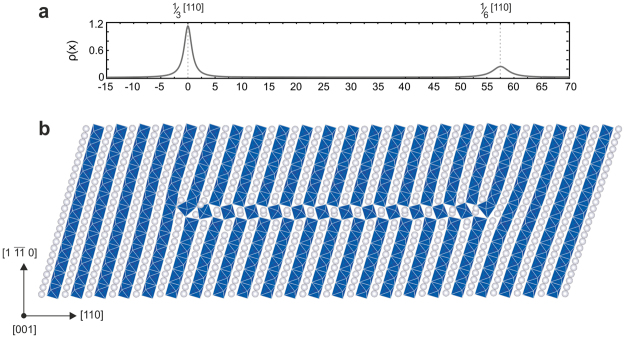
½[110]($1\bar{1}0$) edge dislocation core in MgSiO_3_ post-perovskite computed at 120 GPa. (**a**) Burgers vector density computed from the differential displacement of atoms in the dislocation core region. (**b**) Atomic structure of the edge dislocation core with a perovskite-like stacking fault bounded by two partial dislocations of 1/6[110] and 1/3[110] Burgers vectors.

Atomistic calculations of the core structures of screw and edge dislocations with ½[110] Burgers vectors (performed at a pressure of 120 GPa to account for the D” layer conditions) indicate their strong tendency to spread, and hence to glide, in {110} planes. Indeed, regardless of the dislocation character, a full ½[110] Burgers vector spontaneously dissociates in {110} into two partial dislocations bounding a stacking fault with the typical perovskite-like octahedral interconnections by corners (Fig. 1b). The measurements of the partial dislocation Burgers vectors show that the two partial dislocations are asymmetric with *b*_*p*_ = 1/6[110] and 1/3[110] (Fig. 1a). The equilibrium distance *R* between the partial dislocations (taken as the distance between the two maximum peaks of the Burgers vector density) is on the order of a few nanometres (Fig. 1a). Resulting from a balance between a repulsive elastic force and an attractive force associated with the fault formation energy, this large equilibrium distance suggests a very low stacking fault energy *γ*_*isf*_ associated with the 1/6<110> {110} fault configuration. The partial dislocations are characterised by different Burgers vectors (*b*_*p*_) and also respond to an applied stress with significantly different behaviours. Here, we describe the case of a dislocation with an initial edge character (the study of an initial screw dislocation leads to the same conclusions and is detailed in the supplementary materials). For a typical simulation cell containing approximately 40,000 atoms (350 Å along the direction of the dislocation core dissociation), we calculate that a shear stress in the range of 2.5–2.8 GPa triggers the expansion or the closure of the stacking fault, but this process is due to the sole displacement of the 1/6[110] partial dislocation (i.e. the second partial dislocation remains immobile). The onset of motion of the 1/6[110] partial dislocation is fairly insensitive to the initial stacking fault width or the investigated atomic system size. The 1/3[110] partial dislocation does not glide unless a high stress is applied. However, the 1/3[110] partial dislocation never glides as a partial dislocation, indeed we compute that the full dislocation first recombines into the compact ½[110] Burgers vector dislocation core (requiring an applied stress of 6.5 GPa for the 57 Å core depicted in Fig. 1) before it can actually further glide at an applied stress of 19 GPa.

**Figure 2:**
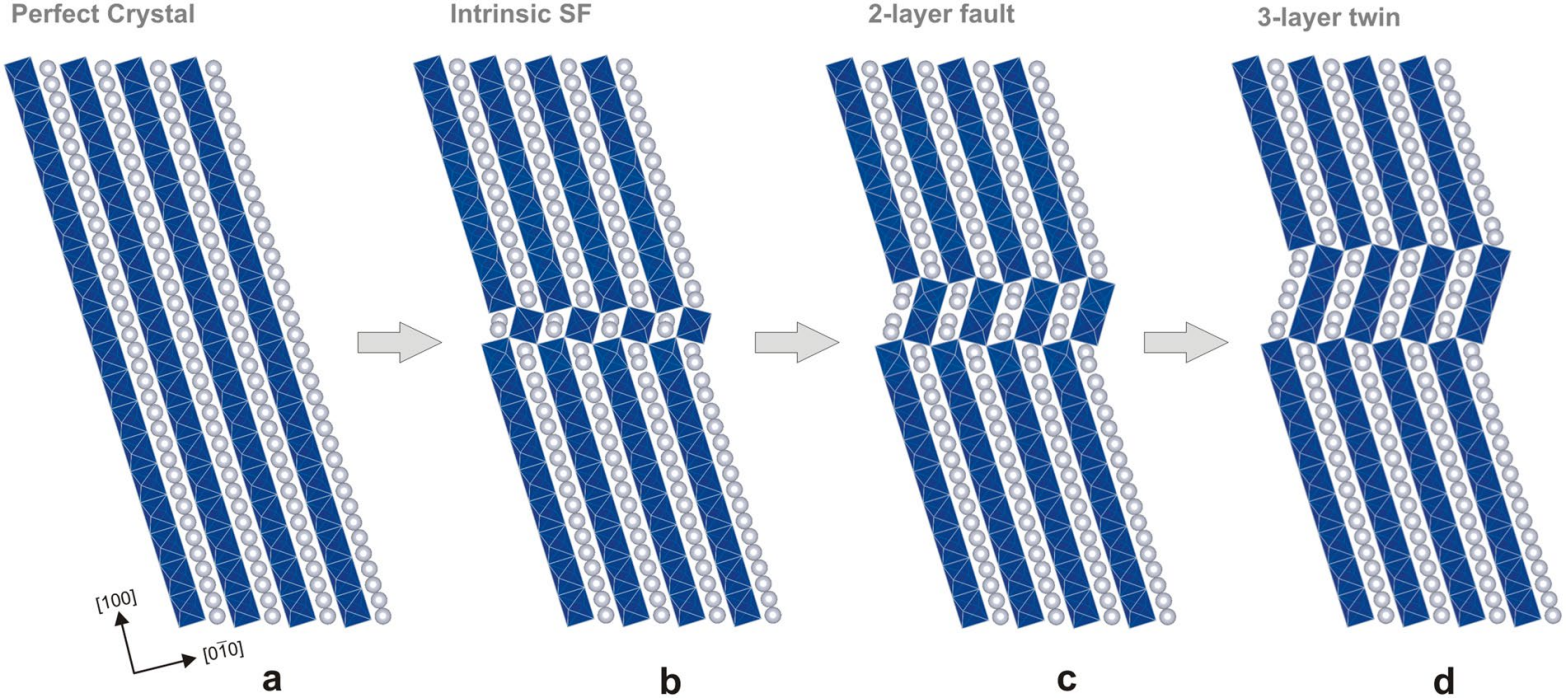
Deformation twinning in ($1\bar{1}0$) with a 1/6[110] partial dislocation of the post-perovskite structure. Starting from the perfect lattice (**a**) viewed along [001], panels (**b**), (**c**) and (**d**) show the lattice with one-, two-, and three-layer faults after the shearing of successive 1/6[110] twinning dislocations. The four atomic structures correspond to the minimum energy configurations in the GPFE landscape of MgSiO_3_ as depicted in Fig. 3.

The conclusions of this study are that neither the [010] nor the ½[110] dislocations are stable or lead to active deformation mechanisms in MgSiO_3_ post-perovskite. Instead, the only dislocation that is mobile is the 1/6[110] partial dislocation, which expands a stacking fault in its wake. Activation of partial dislocation is a deformation mechanism, known to occur in some materials (e.g., Si^22^ and SiC^23^), but that may also lead to another mechanism: mechanical twinning. Indeed, because dislocation glide and deformation twinning are complementary mechanisms potentially contributing to plastic deformation, the occurrence of the highly mobile 1/6<110> partial dislocation raises the possibility of a mechanical twinning mechanism associated with the ½<110> {110} slip system in MgSiO_3_ post-perovskite at 120 GPa. Figure 2 shows that if 1/6[110] partial dislocations are emitted and glide into successive planes, the characteristic structure of the stacking fault leads to the formation of a domain, which appears to be a twin of the orthorhombic structure. To the best of our knowledge, the occurrence of twinning has never been established in MgSiO_3_ post-perovskite, although, interestingly, {110} twin domains have been detected in deformed CaIrO_3_ post-perovskite samples quenched to ambient pressure^13,14^. These experimental observations in this analogue material, which is stable at ambient pressure, provide additional motivation for investigating ½<110> {110} deformation twinning in MgSiO_3_ post-perovskite under pressure conditions corresponding to the D” layer. Because great care must be taken with plasticity mechanism interpretations based on analogues^24^, we conducted calculations for both phases (CaIrO_3_ and MgSiO_3_ post-perovskites) to assess a possible bias (for instance, whether mechanical twinning might readily occur in CaIrO_3_ post-perovskite but not in MgSiO_3_ post-perovskite).

### Twinning mechanism and its associated energy landscape

Deformation twinning is a deformation mechanism that is much more complex to describe than dislocation slip. Let us first consider the nucleation of a 1/6[110] partial dislocation (later called twinning dislocation) in the ($1\bar{1}0$) plane. The *b*_*p*_ = 1/6[110] shear displacement in ($1\bar{1}0$) creates the intrinsic stacking fault (labelled *isf*), forming a one-octahedron-thick perovskite-like lamella (Fig. 2b) similar to that observed in ½<110> {110} dislocation cores (Fig. 1). If this event is followed by further successive nucleation and gliding of similar twinning dislocations in parallel successive {110} planes (Fig. 2c and d), a micro twin is formed. Once the {110} deformation twin lamella is nucleated, it can grow thicker by further activation of twinning dislocations on top of the twinning walls. The geometry of <110> {110} deformation twinning in post-perovskites can be described as the rotation of the parent lattice along the [001] axis by 34.5° in MgSiO_3_ and by 31.1° in CaIrO_3_.

**Figure 3:**
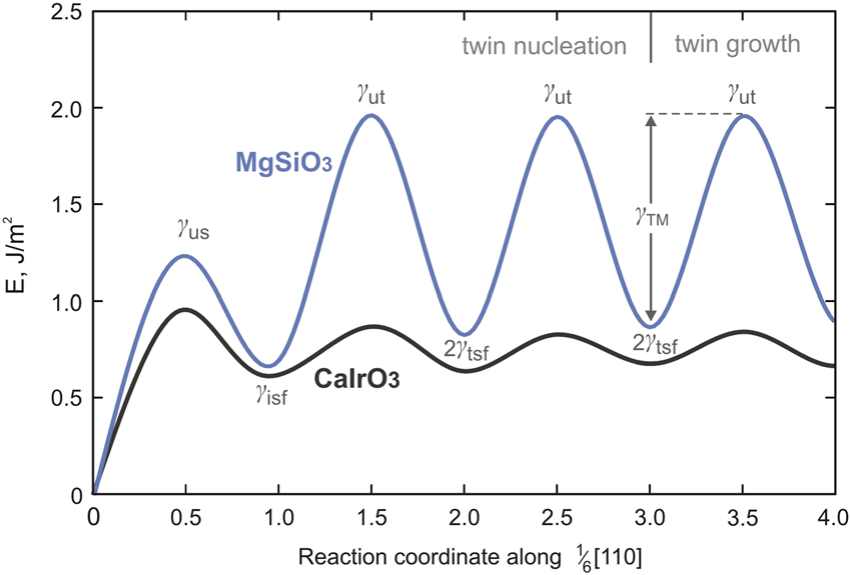
GPFE landscape for <110>{110} deformation twinning in MgSiO_3_ and CaIrO_3_ post-perovskites characterized by *b*_*p*_ = 1/6<110>. The calculated fault energies are given in Table 1.

In this work, we compute the twinning energy landscape (Fig. 3) corresponding to the twin formation mechanism described in Fig. 2 for the two post-perovskite compounds. This energy, also called generalized planar fault energy (GPFE)^25^, corresponds to the cost per unit area required to form a *N*-layer twin by shearing *N* consecutive atomic layers along the [110] direction in the ($1\bar{1}0$) plane. The GPFE first involves the *γ*_*us*_ barrier against a one-layer partial fault becoming a one-layer full fault. This barrier is followed by the one-layer intrinsic stacking fault energy *γ*_*isf*_ (Fig. 2b). Nucleation of the second, third and subsequent 1/6[110] dislocations creates the two-, three- and further *N*-layer stacking faults (Fig. 2c and d for instance). By analogy, the energy barrier opposed to the formation of each subsequent *N*-layer fault (*N* > 1), i.e., the barrier preventing a *N*-layer partial fault from becoming a *N* + 1-layer partial fault, is denoted *γ*_*ut*_, and the energy minimum, 2*γ*_*tsf*_, is twice the energy of the twin stacking fault (i.e., it accounts for the upper and lower twin boundary). The energy difference between *γ*_*ut*_ and 2*γ*_*tsf*_ defines the so-called twin migration energy *γ*_*TM*_^25,26^. As previously discussed by Kibey *et al*.^27^ and Wang *et al*.^28^, the pathway barriers *γ*_*us*_, *γ*_*ut*_ and *γ*_*TM*_ (Table 1) cannot be measured experimentally but nonetheless represent important parameters that strongly affect the critical twin nucleation stress *σ*_*cr*_ as later described. For the investigated <110> {110} twinning system in MgSiO_3_ and CaIrO_3_ post-perovskites, the convergence in *γ*_*TM*_ energy is reached after nucleation of the third twinning partial dislocation, thus resulting in total shear displacement by a full ½[110] lattice repeat. Hence, further nucleation and propagation of successive 1/6[110] dislocations enable twin growth on the developed three-layer twin lamella.

**Table 1:** Characteristic parameters of ½<110>{110} deformation twinning in MgSiO_3_ post-perovskite at 120 GPa and CaIrO_3_ at 0 GPa.

	MgSiO_3_	CaIrO_3_
*μ* (GPa)	327	60
*b*_*p*_ (Å)	1.41	1.73
*ζ* (Å)	3.2	3.9
*s* = *b*_*p*_/*Δh*	0.60	0.57
*γ*_*us*_ (J/m^2^)	1.23	0.96
*γ*_*isf*_ (J/m^2^)	0.69	0.62
*γ*_*ut*_ (J/m^2^)	1.95	0.84
2*γ*_*tsf*_ (J/m^2^)	0.86	0.69
*γ*_*TM*_ (J/m^2^)	1.09	0.15

For the interpretation of the different parameters and energies, the reader is invited to refer to the Results section.

### Twinning stress mechanical model

**Figure 4:**
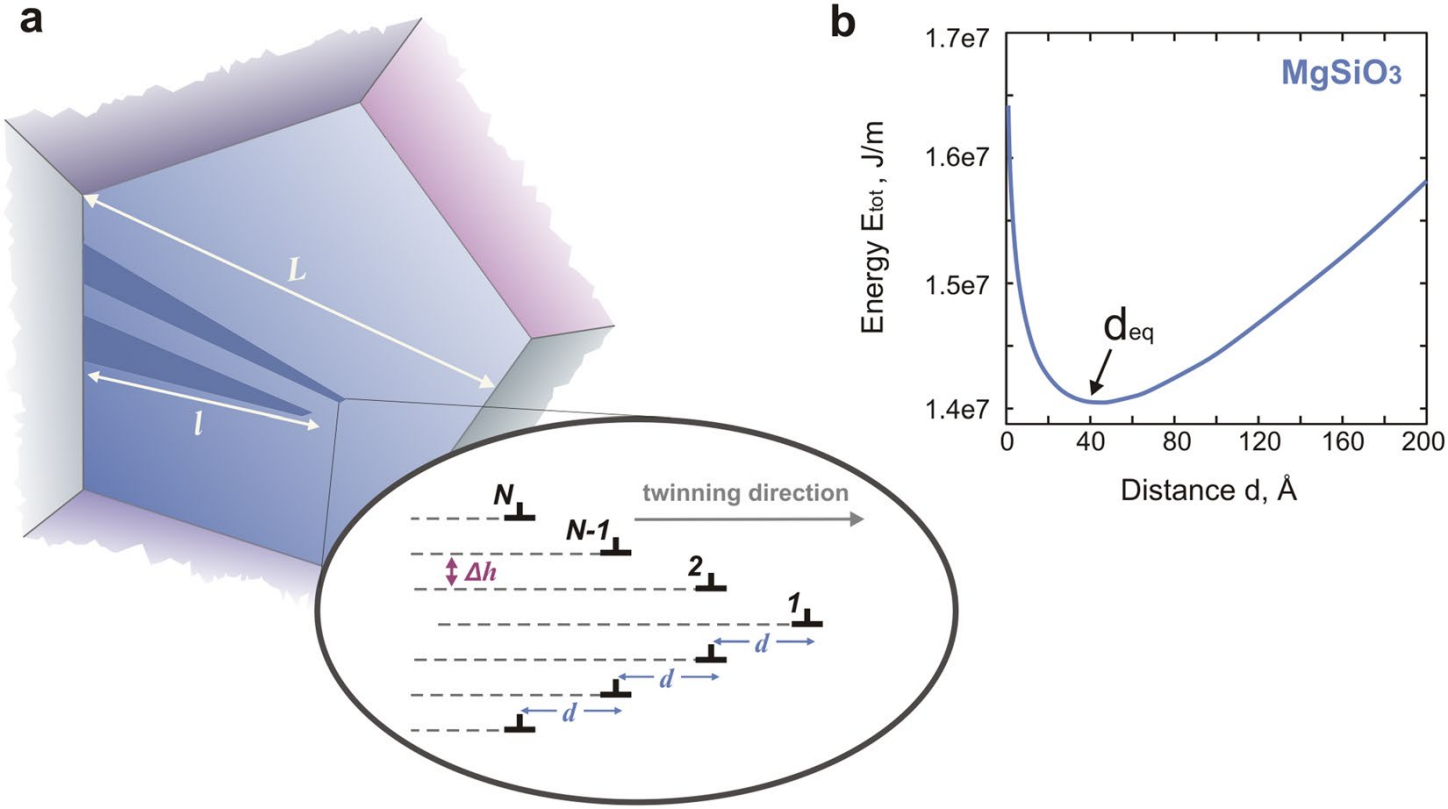
(**a**) Schematic illustration of a semi-lenticular twin morphology characterized by thickness *h*, length *l* and invariant spacing *d* between the neighbouring twinning dislocations. The twinning plane is ($1\bar{1}0$), and the twinning direction is [110]. (**b**) Evolution of the twin energy as a function of the spacing *d* between twinning dislocations. Under a typical applied stress of 100 MPa, the equilibrium distance of twin nuclei is approximately 40 Å.

Considering that deformation twins usually form as individual thin plates embedded in the matrix or in contact with a free surface or a grain boundary^29^, a deformation twin can be described as a series of loops or half-loops of twinning dislocations (Fig. 4a) belonging to twinning planes equidistant from each other by *Δh*. After it is fully nucleated, such a twin lens grows thicker (in the direction normal to the twinning plane) through successive nucleation of new dislocation loops. This thickening (growth) stage is generally easier than nucleation^30^. The total energy associated with the process of twin nucleation^31^ can be defined as follows:

1 $${E}_{tot}={E}_{int}+{E}_{GPFE}+{E}_{l}-W$$

where *E*_*GPFE*_ is the twin boundary energy^28^; *E*_*int*_ is the energy term resulting from the interaction of twinning dislocations; *E*_*l*_ is the twinning dislocation line energy; and *W* is the work of the applied stress. In this equation, the energy term *E*_*l*_ is independent of the spacing *d* between the twinning dislocation, and it consequently does not contribute to the critical twin nucleation stress *σ*_*cr*_ and will be therefore disregarded in the following.

Being characterized by large twinning shear values (*s* = *b*_*p*_ */Δh*) close to 0.6, twin domains in MgSiO_3_ and CaIrO_3_ post-perovskites (Table 1) are very thin relative to their length. As shown by Cooper^31^, the total energy of an extended twin lamella of thickness *h* and half-length *l*, such that *l* ≫ *h*, can be considered as the energy of two flat surfaces containing twinning dislocations, *i.e*., the contribution of the {110} interplanar spacing *Δh* (2.41 Å and 3.06 Å in MgSiO_3_ and CaIrO_3_, respectively) versus the distance *d* between twinning dislocations can be neglected. In such a twin lamella, the twinning dislocations are assumed to belong to two flat surfaces and the total interaction energy of all twinning dislocation^28,31^ is given by:

2 $${E}_{int}=\frac{\mu {b}_{p}^{2}\,}{2\pi (1-\upsilon )}\{\,{N}^{2}{\rm{l}}{\rm{n}}\frac{L}{d}-\,{\rm{l}}{\rm{n}}(N-2)!+\sum _{i=2}^{N-1}[{\rm{l}}{\rm{n}}(N-i)!+\,{\rm{l}}{\rm{n}}(i-1)!\,]\}$$

where *N* is the number of twinning dislocations of Burgers vector *b*_*p*_; *L* is the arbitrary size of a crystal grain (Fig. 4a); ν is the Poisson ratio; *μ* is the anisotropic shear modulus; and *d* is the distance between the *i* ^*th*^ and the (*i* + 1)^th^ twinning dislocations (Fig. 4a), which is assumed to be invariant in this study.

The twin boundary energy *E*_*GPFE*_ consists of two contributions: *γ*_*SF*_, the energy required to create the intrinsic stacking fault, and *γ*_*twin*_, the energy required to nucleate a twin. Relying on the computed characteristics of the GPFE landscape (Fig. 3) and on the fact that twinning dislocations may have classical properties of ordinary dislocations, these two terms can be defined as follows:

3 $$\begin{array}{cccc} & {\gamma }_{SF} & = & {\gamma }_{isf}+({\textstyle \tfrac{{\gamma }_{us}-{\gamma }_{isf}}{2}})\,[1-\,\cos (2\pi {\textstyle \tfrac{f(y)}{{b}_{p}}})]\\  & {\gamma }_{twin} & = & ({\textstyle \tfrac{2{\gamma }_{tsf}\,+{\gamma }_{isf}}{2}})+{\textstyle \tfrac{1}{2}}({\gamma }_{ut}-{\textstyle \tfrac{2{\gamma }_{tsf}+{\gamma }_{isf}}{2}})\,[1-\,\cos (2\pi {\textstyle \tfrac{f(y)}{{b}_{p}}})]\\ {\rm{w}}{\rm{i}}{\rm{t}}{\rm{h}} & f(y) & = & {\textstyle \tfrac{{b}_{p}}{2}}+{\textstyle \tfrac{{b}_{p}}{N\pi }}[\arctan ({\textstyle \tfrac{y}{\zeta }})+\arctan ({\textstyle \tfrac{y-d}{\zeta }})+\cdots +\arctan ({\textstyle \tfrac{y-(N-1)d}{\zeta }})]\end{array}$$

where *f*(*y*) describes the disregistry function of *N* twinning dislocations characterized by the uniform half-width *ζ* and separated from each other by the invariant distance *d* (Fig. 4a). For MgSiO_3_, the half-width *ζ* = 3.2 Å can be directly extracted from the geometric characteristics of the 1/6[110] partial dislocation (Fig. 1), whereas for CaIrO_3_, we use *ζ* = 3.9 Å, accounting for the scaling factor *b*_*p*_(CaIrO_3_)/*b*_*p*_(MgSiO_3_). For *γ*_*SF*_, *f*(*y*) is considered to be in the range 0 ≤ *f*(*y*) ≤ *b*_*p*_, corresponding to the intrinsic stacking fault region, while *γ*_*twin*_ is computed for *b*_*p*_ < *f*(*y*) ≤ *Nb*_*p*_ with *N* > 1, describing the disregistry function of the twin domain nucleated on the existing stacking fault configuration.

Relying on the defined analytic expressions for *γ*_*SF*_ and *γ*_*twin*_ (Eq. 3), the twin boundary energy can be computed as:

4 $${E}_{GPFE}(d)={\int }_{0}^{d}{\gamma }_{{\rm{S}}{\rm{F}}}dy+(N-1){\int }_{0}^{d}{\gamma }_{twin}dy\,$$

Assuming the applied shear stress *σ*_*a*_ to be uniform within the twin domain, the work of the applied stress can be expressed as follows:

5 $$W{=s\sigma }_{a}hl=2{N}^{2}{\sigma }_{a}{b}_{p}d$$

Relying on the energy terms described above (and reported in Table 1), the total energy of a twin lamella can be represented as the function of the spacing *d* between the twinning dislocations (Fig. 4b). The minimum energy configuration corresponds to the equilibrium distance *d*_*eq*_, which increases slightly with applied stress. Thus, for the applied stress in the range of 20–200 MPa, *d*_*eq*_ varies from 39 Å to 50 Å and from 12 Å to 18 Å in MgSiO_3_ and CaIrO_3_, respectively. According to these geometric parameters, the ratio *h*/*l* of a twin lamella in the considered post-perovskites is on the order of 10^−2^.

Finally, the critical twin nucleation stress *σ*_*cr*_ can be further defined while minimizing *E*_*tot*_ (Eq. 1) with respect to the distance *d*: *∂E*_*tot*_/*∂d = *0. Using *N* = 3 defined from the GPFE calculations, we obtain *σ*_*cr*_ values of ~880 MPa and ~620 MPa (see Supplementary Fig. 3) for the <110> {110} twinning system in MgSiO_3_ and CaIrO_3_ post-perovskites.

## Discussion

At the microscopic scale, plastic deformation can result from several mechanisms. In this study we focus on intracrystalline mechanisms. Owing to the very peculiar crystal structure of post-perovskite, dislocation glide appears easy only in the plane of structural layering^19–21^. However, which deformation mechanism effectively accounts for strain components out of the (010) plane remains an open question which is fundamental to address in order to model CPO fabrics and the resulting seismic properties. The fact that [010] and ½[110] dislocations spontaneously decompose into partial dislocations and that only one of these is able to move raises a fundamental question: which mechanism mediates plastic strain out of the (010) plane? Is it plastic slip of either perfect or partial dislocations, or is it mechanical twinning? These three mechanisms involve the emission of a leading 1/6[110] partial dislocation as the first step. In case of full dislocation slip, this first event (*i.e*., the nucleation of a leading partial dislocation) must be followed by the nucleation of the associated trailing 1/3[110] partial dislocation. In case of twinning, the nucleation of the first 1/6[110] partial dislocation is followed by the nucleation of a second 1/6[110] adjacent twinning dislocation. This process may or may not occur, because it may be more favourable to nucleate a second partial dislocation at a completely different location in the crystal rather than next to the first one, thus suggesting that extended partial slip may be a third possible strain-producing mechanism.

According to the work of Rice^32^, the emission of a partial dislocation can be associated with the energy barrier *γ*_*us*_ (Fig. 3 or Supplementary Fig. 2), which a partial dislocation must overcome in order to nucleate. Because twin formation requires at least the nucleation of a second twinning dislocation on a plane where a stacking fault already exists, similarly to Rice approach, Tadmor & Hai^33^ have defined the energy barrier that a twinning partial dislocation must overcome as (*γ*_*ut*_ - *γ*_*isf*_). For full dislocation slip, by analogy, an energy barrier can also be associated with the nucleation of the trailing partial dislocation by considering the difference between *γ*_*isf*_ and the unstable energy corresponding to the trailing partial dislocation. Classically, in fcc metals, the emission of the trailing partial dislocation (which is identical to the leading one) involves thus the sole term (*γ*_*us*_ - *γ*_*isf*_). The situation is different here for post-perovskite compounds, in which the trailing partial dislocation corresponds to a different Burgers vector. The emission of the trailing partial dislocation of partial Burgers vector 1/3<110>, which is basically not mobile, involves a second unstable energy *γ*_*us*_^1*/3[110]*^ that is higher than *γ*_*us*_ (ca. greater than 4 J/m^2^ in MgSiO_3_, see Supplementary Fig. 2).

The competition between the three distinct mechanisms can thus be inferred by considering the different energy barriers associated with the onset of each mechanism (Table 2). Consequently, for the two post-perovskite phases, full dislocation slip is rather unlikely, because it requires overcoming the highest barrier (*γ*_*us*_^*1/3[110]*^ - *γ*_*isf*_) (Table 2), which is higher than the single *γ*_*us*_. This conclusion is also consistent with our calculations of the lattice friction in MgSiO_3_ post-perovskite presented in the first part of the results section. Indeed, calculation of the minimum stress for full dislocation glide yields a stress of 19 GPa. The glide of the full ½<110> dislocation is therefore very unlikely, and the occurrence of such a deformation mechanism in MgSiO_3_ can be ruled out from both an energetic nucleation aspect and from consideration of the lattice friction.

**Table 2:** Calculated barrier energy for the three different plastic strain-producing mechanisms: individual partial dislocation slip, twinning and perfect dislocation slip.

	MgSiO_3_	CaIrO_3_
Emission of 1/6[110] partial dislocation, *γ*_*us*_ (J/m^2^)	1.23	0.96
Emission of twinning dislocation, *γ*_*ut*_ - *γ*_*isf*_ (J/m^2^)	1.26	0.22
Emission of full ½<110> dislocation, *γ*_*ut*_^*1/3[110]*^ - *γ*_*isf*_ (J/m^2^)	>3	>1

The competition between the slip of individual partial dislocations and twinning can be assessed by comparing (*γ*_*ut*_ - *γ*_*isf*_) and *γ*_*us*_ (Table 2). For CaIrO_3_ post-perovskite, the energy barrier that a twinning partial dislocation must overcome is clearly smaller than *γ*_*us*_. Consequently, deformation twinning is expected to be highly favoured in this compound, in agreement with the observation of {110} twin domains in CaIrO_3_ post-perovskite deformed experimentally^13,14^. Additionally, long stacking fault ribbons (resulting from slip of partial dislocations) have never been reported in TEM observations of deformed post-perovskite analogues. The situation is not so straightforward in MgSiO_3_ post-perovskite, because (*γ*_*ut*_ - *γ*_*isf*_) is comparable to *γ*_*us*_. In that case, the simple consideration of *γ*_*ut*_/*γ*_*isf*_ and *γ*_*us*_/*γ*_*isf*_ ratios is not sufficient for robust conclusions^34^. The twinning mechanism efficiency is known to result from a complex interplay between the various energies associated with the GPFE energy landscape, as pointed out by Kibey *et al*.^27^ in fcc Cu-Al alloys. Going beyond these simple energetic considerations is the goal of the hierarchical model described above, which computes the twinning stress on the basis of the entire GPFE landscape. For the MgSiO_3_ post-perovskite phase, the critical twinning stress *σ*_*cr*_ is approximately 880 MPa, computed according to the GPFE calculated at a confining pressure of 120 GPa. By itself, *σ*_*cr*_ already appears to be a rather low value. This value can be further compared to the lattice friction opposed to the motion of a 1/6<110> partial dislocation (for the matter of comparison, we define the lattice friction as the computed minimum stress that must be applied to observe an expansion or a reduction in the stacking fault ribbon if the 1/6<110> partial core is forced to move under an applied shear stress). By computing the ½<110> dislocation core structure and the evolution of the core under applied shear stress, we find that the motion of the leading partial dislocation can occur only at a stress level of 2.7 GPa, which is 3 times higher than the critical twinning stress. In a deforming grain of MgSiO_3_ post-perovskite, the twinning stress will be reached well before the lattice friction of partial slip is overcome. This suggests a high ability for MgSiO_3_ post-perovskite (as in CaIrO_3_) to readily exhibit mechanical twinning, according to the present mechanism involving <110>{110} slip system.

Finally, the twinning stress, computed here at 0 K and supposed to be mostly athermal^30^, can be compared to the lattice friction of dislocation glide in post-perovskite materials^20,21,24^. In CaIrO_3_, the twinning stress reported here is at least 30% below any lattice friction level^24^. For the MgSiO_3_ post-perovskite, it turns out that the twinning stress is also below the stress level of lattice friction for the easiest slip systems since the Peierls stresses for [100](010)^20^ and [001](010)^21^ are 2 and 3 GPa, respectively. Thus, twinning appears to be the best candidate to account for the, so far, missing mechanism responsible for shear along the [010] direction. Up to now, such twinning mechanism has never been considered to explain CPO development in post-perovskite deformation, although the contribution of deformation twinning is especially important in low-symmetry crystals in which information on five independent slip systems is needed to describe a general deformation of the material.

## Methods

### First-principles calculations of GPFE

Generalized planar fault energy (GPFE) calculations were performed based on the density-functional theory (DFT) within the generalized gradient approximation (GGA), with the PW91 parameterisation^35^ and the all-electron projector augment-wave (PAW) method as implemented in VASP^36,37^. To achieve computational convergence, a plane-wave cut-off *E*_*cut*_ of 600 eV was applied. The first Brillouin zone was sampled using the Monkhorst-Pack scheme^38^ with an 8 × 6 × 1 *k*-point grid. The convergence energy is 10^−3^ meV/atom. Simulations were performed at constant volume, corresponding to a bulk volume under confining pressure of 120 GPa and 0 GPa for MgSiO_3_ and CaIrO_3_, respectively. All calculations were performed using fully periodic atomic arrays containing 180 atoms. The simulation cells were built on vectors *a*_*1*_ = ½[110], *a*_2_ = [001] and *a*_3_ = [$1\,\overline{11}\,0$] and oriented in such a way that the twinning direction ½[110] was aligned with the Cartesian *x* axis and that the ($1\bar{1}0$) twinning plane was normal to *z*. Indeed, *a*_3_ is nearly normal (89.1°) to the twinning plane. Along the *z* axis, the simulation cells were as thick as 18 octahedral SiO_6_/IrO_6_ layers, *i.e*., 43.34 Å for MgSiO_3_ and 54.99 Å for CaIrO_3_. The interplanar distance *Δh* between the subsequent {110} stacking planes was equal the size of one SiO_6_/IrO_6_ octahedron along *z*.

To compute the energy landscape, we create the first layer fault by incrementally shift half of the supercell by a twinning Burgers vector *b*_*p*_. For the second layer fault, a new shear level is defined one octahedron above the already existing first layer fault. Atoms above this new level are rigidly shifted by the same incremental amount to reach a total displacement of 2 *b*_*p*_. A similar procedure is repeated to create the third, fourth, *etc* layers. In order to keep the periodicity of the atomic array, the *a*_3_ vector is tilted by the displacement vector. Practically, the path was sampled using eleven shear steps per twinning Burgers vector *b*_*p*_. The full GPFE curve was initially computed using the classical molecular statics simulations described below using pairwise potential of Buckingham form. After this first minimization, we selected four shear steps per twinning Burgers vector with increment 0.25 *b*_*p*_. These configurations were relaxed using the VASP code. For the minimum energy configurations on GPFE curves (resulting from the shear by *N* × *b*_*p*,_ where *N* is a positive integer), full atomic relaxation, including the directions normal and parallel to the shear plane, was allowed. For the high-energy configurations, the degrees of freedom along the ($1\bar{1}0$) plane were restricted only for the Si/Ir sublattice. To ensure optimum geometry of the faulted structure, additional shuffling across the boundary plane was allowed, *i.e*., all the atoms within one octahedral layer right above and below each newly introduced stacking plane (indicated with small black arrows in Supplementary Fig. 2b) were allowed to relax fully.

### Molecular static calculations of the ½<110>{110} dislocation core structure in MgSiO_3_ post-perovskite

For MgSiO_3_ post-perovskite, atomistic simulations of dislocation cores at 120 GPa were performed using classical molecular statics (MS) simulations. The force field used corresponds to the Buckingham form of a pairwise potential parameterized by Oganov *et al*.^39^. Calculations were performed using the LAMMPS package, relying on Ewald summation methods for Coulombic interactions^40^. Optimization of the dislocation core configurations was performed using a conjugate gradient minimizer on a so-called periodic slab geometry containing a single dislocation core, as described by Hirel *et al*.^41^. The simulation cells were designed to be fully periodic along the dislocation line direction (Cartesian *x* aligned with ½[110] for screw dislocations and with [001] for edge dislocations) and the dislocation glide direction (Cartesian *y* aligned with [001] for screw dislocations and with ½[110] for edge dislocations). Along the direction normal to the {110} glide plane (Cartesian *z*), atoms at the bottom and at the top of the supercell were kept fixed to their regular positions given by the dislocation long-range elastic field. The total width (along *z*) of the layer with fixed atoms was equal to the potential short range cut-off distance of 12 Å. For screw dislocations, the supercell parameter aligned with *y* was tilted by ½*b* along *x* to maintain periodicity along the dislocation glide direction. The simulation cell size was equal to one unit cell repeat along *x*, 160 Å along *z* and varies from 300 to 450 Å along *y*. Such simulation systems contained approximately 36,000–70,000 atoms. To initiate a dislocation glide, *ε*_*xz*_ and *ε*_*xy*_ strain components were gradually increased for screw and edge dislocations, respectively. To ensure quasi-static loading, the sheared atomic configuration was optimized every shear strain increment of 10^−5^–10^−4^.

## Electronic supplementary material

Supplementary material
