# Supplementary material for: Prediction of Mechanical Twinning in Magnesium Silicate Post-Perovskite
Source: Sci Rep. 2017 Dec 15;7:17640. doi: 10.1038/s41598-017-18018-1 (PMC5732224; doi:10.1038/s41598-017-18018-1)
Supplement: Supplementary file 1 — Supplementary material [file 41598_2017_18018_MOESM1_ESM.pdf]

SUPPLEMENTARY MATERIALS

Prediction of Mechanical Twinning in Magnesium Silicate Post-Perovskite

Philippe Carrez*, Alexandra M. Goryaeva, and Patrick Cordier

UMET-Unité Matériaux et Transformations
CNRS, INRA, ENSCL, UMR 8207
Université de Lille, 59000 Lille, France

* corresponding author: philippe.carrez@univ-lille1.fr

Supplementary Figure 1: ½<110>{110} screw dislocation core structure

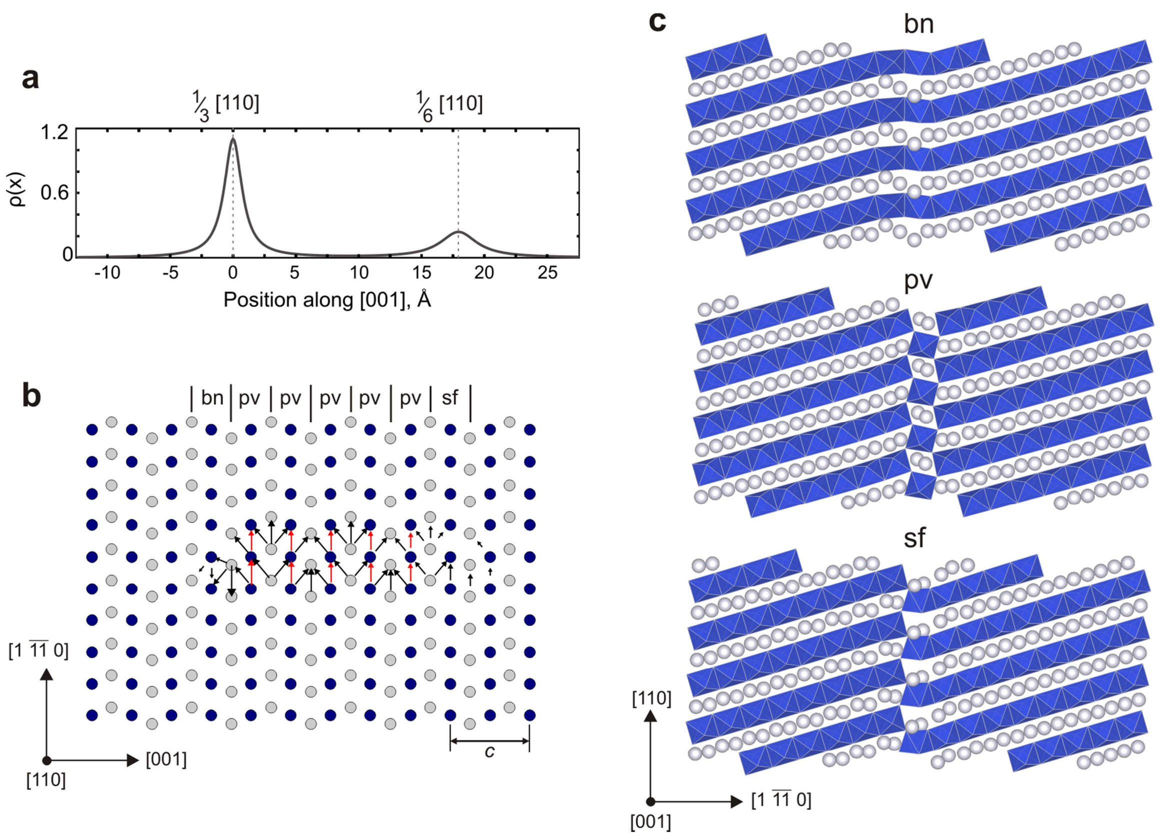

(a) Burgers vector density of the relaxed ½<110>{110} screw dislocation core. **(b)** Differential displacement map of the relaxed ½<110>{110} screw dislocation core. The anion sublattice is left out. Si atoms are shown with blue balls; Mg atoms—with grey balls. The arrows between atoms correspond to the ½[110] component of the relative displacement of the neighbouring atoms produced by the dislocation. Red arrows indicate interconnection by corners of Si-octahedra. **(c)** Atomic structure of the three distinct {001} atomic layers, denoted as ‘bn’, ‘pv’ and ‘sf’. Location of these layers in the core structure is specified in (b)

Supplementary Figure 2: Generalized stacking fault calculations for ½<110> {110} system

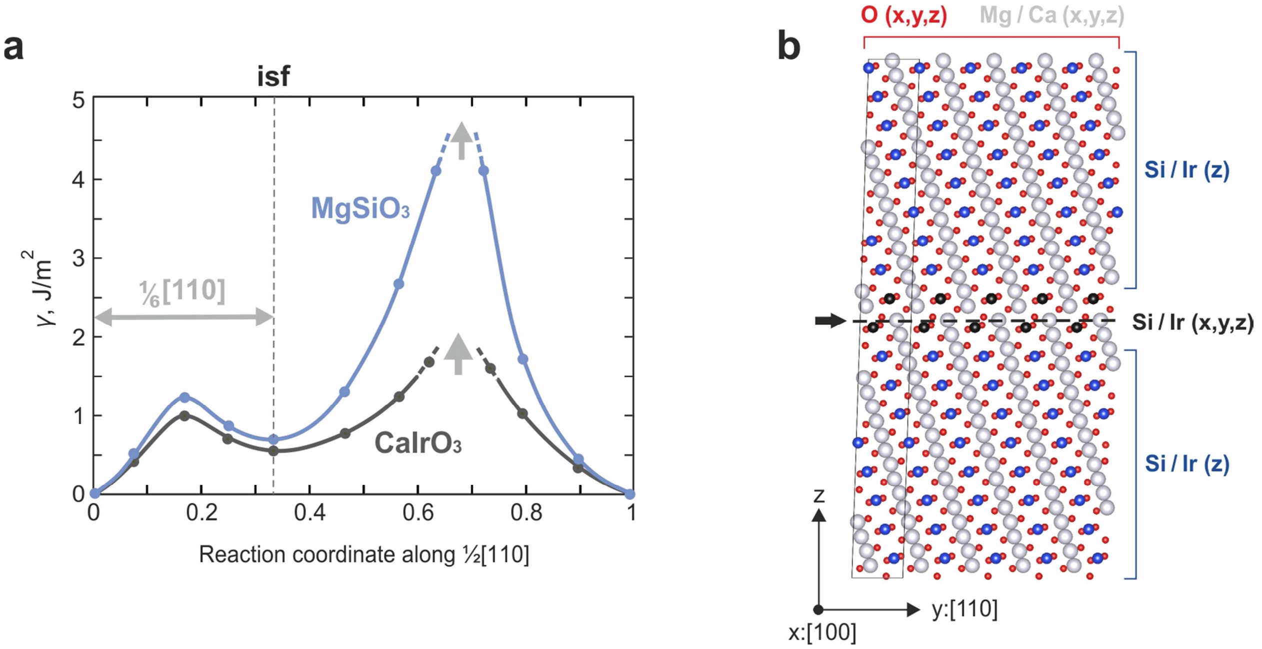

(**a**) ½<110>{110} *γ*-energies computed for $MgSiO_3$ and $CaIrO_3$ according to an optimized atomic relaxation conditions, as depicted on **(b)**. Compared to conventional relaxation scheme (all the atoms are only allowed to relax along the direction normal to the shear plane) we use a 'mixed' relaxation scheme for ½<110>{110} system in the post-perovskite that allows optimizing the stacking fault configuration without annihilating applied rigid shear. Optimum configuration of the stacking fault is reached while allowing all the 3 sublattices in contact with stacking plane to relax fully, *i.e.* including the (*x*, *y*, *z*) directions normal and parallel to the stacking plane. In order to ensure rigid shear in the crystal, degrees of freedom along the shear plane are restricted for all the Si/Ir atoms, which are not in contact with the stacking plane. The resulting excess energy curves (a) are characterized by the apparent local energy minimum at $^1/_6$ [110] shear, indicating ½<110> → $^1/_6$ <110> + $^1/_3$ <110> dissociation in {110} plane. The employed atomic relaxation scheme however cannot fully handle the calculations of unstable stacking fault energies $\gamma_{us}^{1/3[110]}$ at $^1/_3$[110] shear as indicating by arrows.

Supplementary Figure 3: Critical twin nucleation stress computed for $MgSiO_3$ and $CaIrO_3$

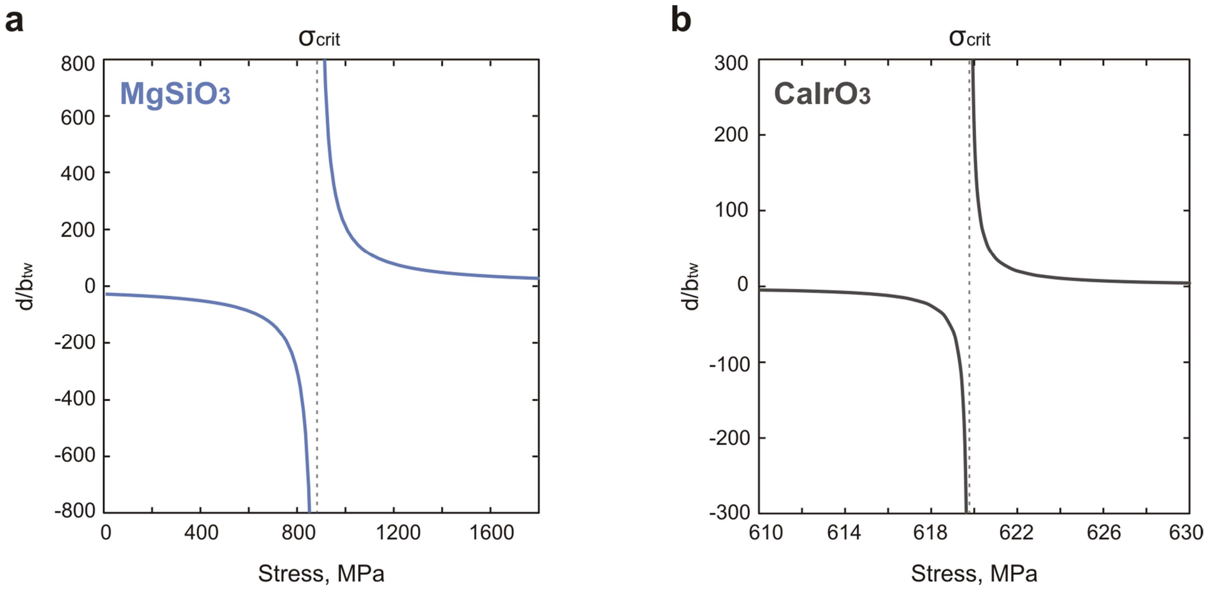

For both compositions, the stress is computed as $\partial E_{tot}/\partial d$ = 0 for the three-layer twin nucleus (N=3).
